# Automatic x‐ray image contrast enhancement based on parameter auto‐optimization

**DOI:** 10.1002/acm2.12172

**Published:** 2017-09-06

**Authors:** Jianfeng Qiu, H. Harold Li, Tiezhi Zhang, Fangfang Ma, Deshan Yang

**Affiliations:** ^1^ Department of Radiology Taishan Medical University Taian China; ^2^ Department of Radiation Oncology Washington University School of Medicine St. Louis MO USA

**Keywords:** image guidance, image processing, radiation therapy

## Abstract

**Purpose:**

Insufficient image contrast associated with radiation therapy daily setup x‐ray images could negatively affect accurate patient treatment setup. We developed a method to perform automatic and user‐independent contrast enhancement on 2D kilo voltage (kV) and megavoltage (MV) x‐ray images. The goal was to provide tissue contrast optimized for each treatment site in order to support accurate patient daily treatment setup and the subsequent offline review.

**Methods:**

The proposed method processes the 2D x‐ray images with an optimized image processing filter chain, which consists of a noise reduction filter and a high‐pass filter followed by a contrast limited adaptive histogram equalization (CLAHE) filter. The most important innovation is to optimize the image processing parameters automatically to determine the required image contrast settings per disease site and imaging modality. Three major parameters controlling the image processing chain, i.e., the Gaussian smoothing weighting factor for the high‐pass filter, the block size, and the clip limiting parameter for the CLAHE filter, were determined automatically using an interior‐point constrained optimization algorithm.

**Results:**

Fifty‐two kV and MV x‐ray images were included in this study. The results were manually evaluated and ranked with scores from 1 (worst, unacceptable) to 5 (significantly better than adequate and visually praise worthy) by physicians and physicists. The average scores for the images processed by the proposed method, the CLAHE, and the best window‐level adjustment were 3.92, 2.83, and 2.27, respectively. The percentage of the processed images received a score of 5 were 48, 29, and 18%, respectively.

**Conclusion:**

The proposed method is able to outperform the standard image contrast adjustment procedures that are currently used in the commercial clinical systems. When the proposed method is implemented in the clinical systems as an automatic image processing filter, it could be useful for allowing quicker and potentially more accurate treatment setup and facilitating the subsequent offline review and verification.

## 1 INTRODUCTION

**Figure 1:**
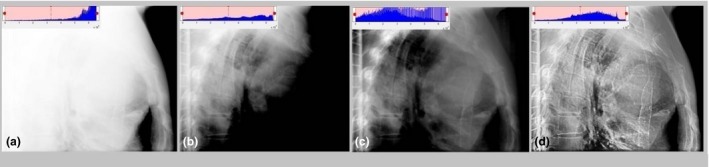
A lateral chest RT image of a lung cancer patient. The spine and rib cage are intended to be used to guide patient setup. (a) Original image in which the spine is invisible because spine's pixel intensity is compressed to 0.14% of the entire pixel intensity dynamic range. (b) Image processed using manually selected optimal windows/level settings. (c) Image processed using CLAHE in which the spine is still not shown well. (d) Image processed using the proposed method showing significantly improved visualization of both the spine and lung.

In image‐guided radiation therapy (IGRT), 2D orthogonal x‐ray images, using either kV or MV, are commonly used to determine the 3D shifts of the treatment couch to align the patient to the correct treatment position in relation to machine isocenter.^1^, ^2^, ^3^, ^4^ However, these images, as shown in Fig. 1, are often associated with poor image contrast and nonuniform image intensity.^5^, ^6^, ^7^, ^8^, ^9^ The onboard imaging system at the treatment console usually only provides basic image processing tools, e.g., windows/level adjustment. While the offline review systems used by the physician and physicist during chart review, e.g., MOSAIQ (Elekta, Stockholm, Sweden), provide additional image filtering options, e.g., AHE (Adaptive Histogram Equalization) and CLAHE (Contrast Limited AHE) to facilitate image reviews, the results are often not satisfactory.

Histogram equalization^10^, ^11^ (HE) with or without adaptive is a relatively simple image processing method to stretch the histogram of the image intensity evenly according to pixel intensity probability.^12^, ^13^ However, HE is not able to avoid high peaks (i.e., clusters of image intensity) in the histogram; therefore cannot enhance the contrast between pixels with the peaks, i.e., within a small range of image intensity. The contrast limited adaptive histogram equalization (CLAHE) algorithm^11^, ^14^ has been developed to overcome such limitations by processing the image histogram in blocks, limiting the intensity dynamic range,^15^ and then clipping and redistributing the gray peaks.^14^, ^16^ CLAHE has been applied to a variety of medical images^17^, ^18^, ^19^, ^20^, ^21^ including mammogram,^22^ digital radiology,^23^ and entropy.^24^ Although more advanced, to achieve optimal results, CLAHE requires user to select several important parameters including block size and contrast limit, which is not automated and thus a time‐consuming trial‐and‐error process. In fact, the CLAHE implementation in MOSAIQ is simple and uses fixed parameters for all images. As such it does not perform well on many 2D x‐ray images, as shown in Fig. 1(c).

The goal of this work was to improve both automation and performance of the use of CLAHE in RT image processing. We hypothesize that, given additional information regarding image acquisition and patient (including treatment site, x‐ray energy, kVp, mAs, and patient size), it is feasible to automate the imaging processing process with significantly improved performance. We note that the patient information can be obtained from the database of the treatment management system while the image acquisition information obtained from the image meta‐data. Here we develop an optimized image processing chain to enhance the image contrast of 2D RT localization images automatically, which consists of a noise reduction filter, a high‐pass filter, and a CLAHE filter. The innovations involved in this study are: (a) to determine the optimal parameters automatically by iteratively maximizing image contrast based on known treatment site and imaging modality and (b) to apply a high‐pass filter before CLAHE to reduce illumination heterogeneity across the entire image and to equalize the regional histogram.

## 2 MATERIALS AND METHODS

### 2.A Workflow

**Figure 2:**
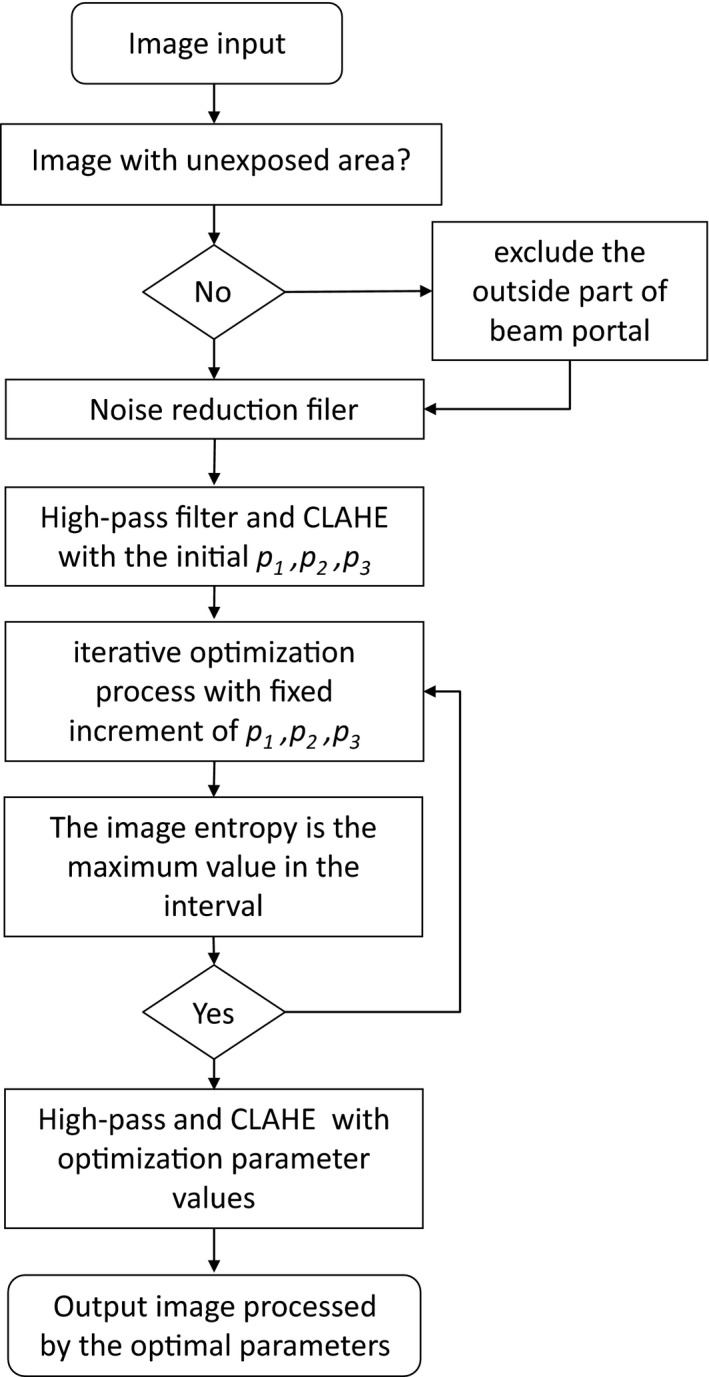
Workflow of the proposed automatic x‐ray contrast enhancement method.

The image processing chain is shown in Fig. 2. The preprocessing step consists of a median filter to reduce image noise, and, for MV images, an additional intensity‐thresholding to detect the beam portal, i.e., only the image pixels inside the beam portal are considered in the subsequent steps.

There are two compelling reasons to use high‐pass filter prior to applying the CLAHE filter: (a) to reduce the image intensity nonuniformity and (b) to enhance the edge of the bony structures. The high‐pass filter is accomplished by subtracting the weighted Gaussian blurred image from the original image:

(1) $$F_{H}=F_{1}-p_{1}G_{\sigma }⊗F_{1},$$

where $F_{1}$ is the input x‐ray image, $F_{H}$ is the high‐pass filtered image, $p_{1}\subset \left[0,1\right]$ is the weighting fact that determines the degree of contour enhancement, $G_{\sigma }$ is the 2D Gaussian kernel, and σ is the Gaussian window width.

The CLAHE filter is then used to equalize the image histogram. CLAHE can avoid gray level peaks associated with HE or AHE by weighting between regional and global histogram equalization. In CLAHE, $p_{2}$ is the number of blocks in X or Y direction of the image, which defines the block size, and $p_{3}$ is the clip limiting parameter, which limits the proportion of the truncated and the histogram peaks which in the every block. $F_{H}$ is segmented into ${p_{2}}^{2}$ blocks^14^, ^25^ and the clipped histogram equalization function is computed per block and then applied on the whole $F_{H}$ by interpolating between neighboring blocks.

### 2.B Optimization

The overall performance of the high‐pass filter followed by the CLAHE filter is significantly affected by the choices of the parameters for the two filters, i.e., the weighting factor $p_{1}$ in the high‐pass filter, the block size $p_{2}$, and the clip limiting parameter $p_{3}$ in the CLAHE method. The optimal values of the three parameters are traditionally determined empirically based on visual assessment over multiple trials. To determine them automatically and quantitatively, we designed an iterative optimization process. The parameters were initialized to a suitable value according to the information available about the patient and the image acquisition, and were then optimized iteratively according to disease site and treatment modality‐dependent objective.

The optimization, which is designed to obtain the maximal entropy in the processed image, can be described as:

(2) $$E\left(p_{1},p_{2},p_{3}\right)=entropy\left(F_{C}\left(F_{H}\left(F_{1}\left(x,y\right),p_{1}\right),p_{2},p_{3}\right)\right.$$

(3) $$\left({\hat{p}}_{1},{\hat{p}}_{2},{\hat{p}}_{3}\right)={\mathrm{argmax}}_{p_{1},p_{2},p_{3}}\left(E\left(p_{1},p_{2},p_{3}\right)\right)$$

where $F_{H}$ is the high‐pass filter, $F_{C}$ is the CLAHE filter, entropy() is the function to compute the image entropy, and ${\hat{p}}_{1},{\hat{p}}_{2},{\hat{p}}_{3}$ are the optimal parameter values. The image contrast is commonly referred to as the intensity difference between the voxels with higher intensity and lower intensity in a local region, while the image entropy is often used to characterize the uncertainty at a system level. Many studies have shown that the image entropy can represent the richness of global image contrast.^23^, ^24^

Finally, the optimal parameters are applied to generate the final contrast‐enhanced image, i.e., the maximal entropy image, as:

(4) $$F_{2}=F_{C}\left(F_{H}\left(F_{1},{\hat{p}}_{1}\right),{\hat{p}}_{2},{\hat{p}}_{3}\right)$$

### 2.C Implementation

The beam portal in an MV image was automatically detected using a simple thresholding method, with a fix threshold value of 50% of the maximal image intensity value. The image pixels in the area outside the MV beam portal were set to null and excluded in the optimization.

Iterative optimization was implemented with an internal point algorithm, which finds the optimum of a nonlinear convex optimization objective by searching the interior of the possible region.^26^ To improve computation speed, the parameters’ initial values and ranges have been determined empirically as listed in Table 1 for each treatment site. For example, the full range of $p_{1}$ was [0, 1]; however, the useful range was [0, 0.85] because the high‐pass filtered image with $p_{1}>0.85$ would be too noisy. Similarly, $p_{2}$ was also limited as an integer in the range of [2, 6]. We note that entropy is subject to image noises and image boundaries, which will cause the value of the entropy to tend to become extreme. However, if we limited the range of the parameter values, the image noise level can be controlled at an acceptable level.

**Table 1:** Empirically determined optimal parameter value range per anatomical site

Imaging position	Weighting factor ($p_{1}$)	Number of blocks (${p_{2}}^{2}$)	Clip limiting ($p_{3}$)
Brain	0.60–0.70	4	0.20–0.30
Head‐neck	0.65–0.70	16	0.45–0.55
Chest posterior–anterior view	0.55–0.65	4	0.35–0.45
Chest lateral view	0.65–0.75	16	0.35–0.45
Spine lateral view	0.70–0.75	4	0.10–0.20
Pelvis lateral view	0.70–0.75	16	0.35–0.45
Spine posterior–anterior view	0.60–0.65	4	0.35–0.45
Pelvis posterior–anterior view	0.55–0.60	16	0.35–0.45
Extremities	0.50–0.55	4	0.20–0.30
Thorax and breast	0.50–0.55	4	0.10–0.20
Shoulder	0.70–0.75	16	0.35–0.45
Pelvis or prostate, with implant marker	0.55–0.65	4	0.35–0.45

The visualization of the bony structures was enhanced with the entropy optimization method. Certain sites, e.g., breast and lung, require the enhancement of the soft tissues, and the pelvis, the implanted metal fiducials. For these sites, the initial values and ranges of the optimization parameters were empirically selected to allow the best contrast of the implants or the soft tissues.

## 3 RESULTS

**Figure 3:**
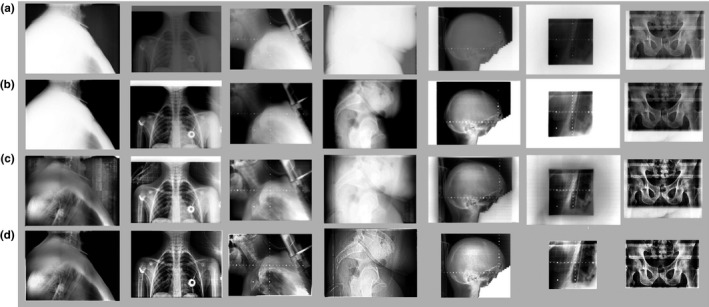
Examples of the processed images. Rows: (a) original images, (b) images processed using optimal windows/level adjustment, (c) images processed using standard CLAHE algorithm, (d) images processed by the proposed method. Columns 1–4 are kV images, and columns 5–7 are MV image. Note that the white borders caused by the treatment beam collimation were auto‐detected and cut‐off in the last two images in row (d).

**Figure 4:**
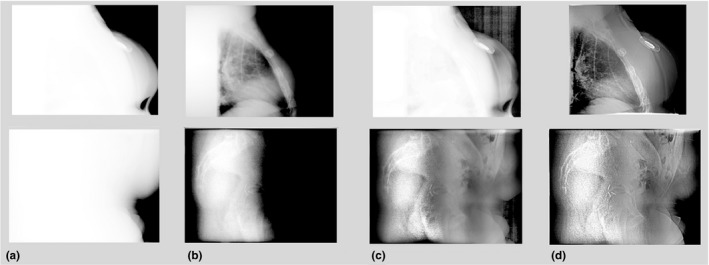
Examples of contrast enhancement of both soft tissue and implant markers. (a) Original images, (b) optimal windows/level setting, (c) standard CLAHE algorithm, and (d) the proposed method.

Total 34 and 18 MV images of patients receiving radiation therapy were included in this study after the images had been anonymized. Anatomical sites included brain, head‐neck, chest, abdomen, and pelvis. Example images are shown in Fig. 3, where the visualization of the bony structures, e.g., the vertebral column and the pelvic bone, has been significantly improved, especially in the areas with high image intensity values. Figure 4 shows two cases for which the images are processed with parameters optimized for visualization of both the soft tissue and the implanted metal markers. The average computation time for each image is 0.78 s.

A blind subjective ranking test was performed to evaluate the proposed method. Fifty‐two original images and 156 images processed using (a) manual windows/level adjustment, (b) standard CLAHE, and (c) the proposed method were visually evaluated and ranked by two physicists and two radiation oncologist with scores of 1 to 5: 1 — worst, unacceptable, 2 — worse than acceptable, barely adequate to support clinical decision, 3 — acceptable, adequate to support clinical decision, 4 — better than adequate, and 5 — significantly better than adequate and visually praiseworthy. The order of the images was randomized so that the observers did not know the corresponding image processing methods. The rank results are listed in Table 2. The mean score of the images processed by the proposed method is 3.92, which is close to a score of 4 (better than adequate) and clearly higher than the mean scores of the other three methods, with *P* values less than 0.0011 based on a Student *t*‐test statistical analysis. The number of unacceptable images was reduced to 10%, less than the number of unacceptable images either unprocessed or processed by other methods. Note that the unacceptable images were all MV portal images. Mainly limited by the imaging modality, the contrast enhancement results of these MV images were ranked *worst, unacceptable* due to either excessive image noise or insufficient contrast between tissues of interests.

**Table 2:** Results of subjective ranking for the processed images

	Score = 1 (%)	Score = 2 (%)	Score = 3 (%)	Score = 4 (%)	Score = 5 (%)	Score mean
Original images	72	14	6	4	4	1.54
Images processed by windows level adjustment	48	20	7	7	18	2.27
Images processed by basic CLAHE	39	13	7	13	29	2.83
Images processed by proposed method	10	8	10	24	48	3.92

## 4 DISCUSSION

The proposed image contrast enhancement method is a fully automatic method after the treatment site information is either manually specified or automatically obtained from the clinical treatment computer systems, e.g., MOSAIQ and ARIA. A machine learning method,^27^ which automatically recognizes anatomical site and image acquisition angle (i.e., view) in the 2D x‐ray images, could also be used as a preprocessing step to obtain the required treatment site and view information. The proposed method combines the advantages of high‐pass edge enhancement and CLAHE to enhance the image contrast automatically. The high‐pass filter enhances structure edges, e.g., edges of the bony structures, which are hidden in the high‐brightness regions, and the subsequent CLAHE filter adaptively extends the range of the image intensity gray levels. The optimal values of the three parameters, $p_{1},{}^{∼}{}^{∼}p_{2}$ and $p_{3}$ are automatically determined using an optimization process.

The x‐ray image acquisition parameters, i.e., kVp, mA, and ms, should be selected optimally by the therapist according to anatomical site, image acquisition angle, patient height and weight so that the quality of the acquired x‐ray images is optimal before the proposed contrast enhancement method is applied. This should be accomplished by training the machine therapists. It would be also useful to define the standard clinical kV image acquisition parameters for different anatomical site and patient size so that the machine therapists can follow.

As we have learned in the preliminary studies, 2D x‐ray images need to be processed differently for different imaging beam orientations (e.g., anterior–posterior and right‐lateral) and disease sites (e.g., brain and pelvis). To allow a quick convergence and optimal results by the optimization process, the site‐dependent initial parameter values and the allowed parameter value ranges have been determined empirically and provided in Table 1. To be fully automated, the proposed method therefore needs two additional pieces of information — treatment site and imaging beam orientation. After the key information is confirmed, the proposed method can be implemented in the image processing workflow of clinical RT systems. In clinical practice, the treatment site could be manually configured by users or automatically obtained using SQL queries from the treatment management system (TMS), e.g., ARIA (Varian Medical, Palo Alto, CA, USA). The imaging beam orientations are usually available in the image DICOM file as imaging beam angles, and are available in the TMS.

## 5 CONCLUSION

We developed a method to automatically enhance the contrast for the 2D x‐ray images used in radiation therapy patient treatments. Our results have shown that this method outperforms basic image processing methods currently used in clinical systems. When the proposed method is implemented in the clinical systems as an automatic image processing filter, it could be useful in many clinical applications including patient treatment setup and subsequent offline review of patient daily setup.

## CONFLICT OF INTEREST

All authors approved the final manuscript, and declared that they have no potential conflicts of interest to this work.
